# No Band Color Effects on Male Courtship Rate or Body Mass in the Zebra Finch: Four Experiments and a Meta-Analysis

**DOI:** 10.1371/journal.pone.0037785

**Published:** 2012-06-07

**Authors:** Aurelie Seguin, Wolfgang Forstmeier

**Affiliations:** Max Planck Institute for Ornithology, Seewiesen, Germany; Arizona State University, United States of America

## Abstract

Replication of experiments is essential for distinguishing real effects from type 1 errors and idiosyncrasies. One of the most replicated experiments in behavioral ecology is the presumed manipulation of male attractiveness in zebra finches by adding red or green color bands. Red-banded males were found to have higher fitness than green-banded males, and most empirical evidence suggests that this effect is mediated by female mating preferences rather than by male-male competition. A recent study, however, reported that color bands affected male courtship rate and body mass independently of female behavior. If this effect was real, some earlier findings of female preferences and maternal effects on offspring traits could potentially be reinterpreted as being mediated indirectly via effects on male behavior. This new perspective seems appealing also in light of a growing interest in bi-directional feedback mechanisms between endocrinology and ornamentation. However, here we report four independent failures to replicate this effect of color bands on courtship rate and body mass. Combining this new experimental data with all the published evidence in a meta-analysis shows that color bands seem to affect neither male courtship rate (average effect size d = 0.02) nor male body mass (d = −0.07). The present case is a reminder that replication of experiments lies at the heart of distinguishing between real effects and false positive findings.

## Introduction

Behavioral ecology research has often been criticized for a lack of replication of important research findings [1], [2], [3]. Replication is important because isolated findings may be misleading for a variety of reasons, e.g. context specificity, errors due to multiple-hypothesis testing [4], flexibility of analysis [5], and all forms of publication and attention bias against null findings [6]. One of the most replicated experiments in behavioral ecology is the putative manipulation of male attractiveness in zebra finches (*Taeniopygia guttata*) by fitting the birds with either red or green color bands. At least 32 published studies report using this technique in zebra finch research (see Table S1), and there are likely many more attempts that have not led to publications (see e.g. [7]).

Several studies found that male zebra finches wearing red bands had higher fitness (production of offspring in communal aviaries) than green-banded males [8], [9], [10]. Most plausibly, this effect is due to sexual selection, so it could come about either (1) because of a female preference for red-banded males or (2) because red-banded males have an advantage in male-male competition, or a combination of these two effects.

1. Support for the first explanation (female preference) comes from choice-chamber experiments [11], [12], [13] and from observations of extra-pair copulations in breeding aviaries [14]. However, not all choice-chamber experiments have revealed such a female preference for red-banded males [15], and it seems likely that such ‘failures’ to replicate often remain unpublished (e.g. own unpublished data). Some additional support for the hypothesis that fitness differences are mediated by a female preference for red-banded males has been inferred from studies of differential investment. Females paired to red-banded males have been found to make greater investments in terms of number of clutches laid ([16], but see [9]), parental care ([17], but see [18]) and testosterone content of egg yolks ([19], but see [20]). However, female zebra finches have also been found to make greater investments for less attractive males (not using color bands) in terms of egg size and quality [21]. Hence, patterns of investment remain difficult to interpret [22], especially due to a lack of consensus in findings and due to uncertainty about the correct *a priori* hypothesis; so the positive evidence for female preferences comes mostly from choice-chamber studies.
2. Support for the second explanation (male-male competition) comes from a study showing that red versus green color bands affected male dominance (supplanting behavior) as well as patterns of diurnal changes in body mass and fat deposition [23]. However, Ratcliffe and Boag [24] found no band color effects, neither on supplanting behavior nor on the males’ ability to defend a nest box. Also, Schuett and Dall [7] found no effects on dominance (supplanting) or body mass.

Hence, there is generally more support for the hypothesis that color band effects on male fitness are mediated by female preferences than by male-male competition. However, a recent study by Pariser et al. [25] seems to shed new light on these relationships. In that study, wild-caught adult male zebra finches were fitted with either red or green bands and were housed in uni-sex groups in aviaries for a period of five months. The study found that red-banded males gained body weight in comparison to green-banded males (longitudinal analysis, i.e. mass after treatment minus mass before treatment), and particularly that red-banded males had a higher courtship rate in experimental encounters with females than green-banded males (cross-sectional analysis after the treatment). Due to the housing in male-only groups there was no feedback from females, hence color bands were suspected to have affected male dominance and thereby condition (with mass and courtship rate being the condition indicators). Since courtship rate has often been related to male attractiveness, at least in some contexts, ([25], [26], [27], [28], but see [29]), it is possible that some of the previously described color band effects may have been mediated indirectly via male behavior and physiology [22].

The issue that manipulation of male ornaments may actually alter more than the females’ perception of male attractiveness, has recently received growing attention [30], [31]. Ornaments may not only be influenced by the carrier’s physiological (e.g. hormonal) state, but may actually also affect physiology due to behavioral interactions with conspecifics ([30], [32]). The findings of Pariser et al. [25] suggest such a feedback from ornamentation to behavior and physiology and therefore the study seems very worth replicating. We here particularly focus on the color band effect on courtship rate, because courtship rate seems to have a high potential to mediate indirect effects. Specifically, it might be that band color affects courtship rate, and courtship rate in turn affects female choice and maternal investment.

Male courtship rate varies greatly between individual males and is highly repeatable within males (repeatability of about 60%, [33]), when measured during 5-minute experimental encounters with unfamiliar females (see [25], [33]). Quantitative genetic analyses indicate that more than half of this repeatable component cannot be explained by either additive genetic variation or maternal effects [34], so there are strong and permanently lasting [29] environmental effects on courtship rate. We hypothesized that these consistent individual differences may be due to long-term effects of experiences gathered during adolescence. Adolescent males may collect information about their own attractiveness (via feedback from females which they court; [32], [35]) and may adjust their own courtship rate strategically in response to that [32], [36]. We expected that males which would wear red bands throughout their entire adolescence would receive positive feedback and acquire higher courtship rates than those wearing green bands [32]. We further expected that this would be a more powerful experimental treatment than the treatment of adult birds (as implemented by Pariser et al. [25]). Treatment of adults targets the behavioral flexibility of fully mature and highly experienced adults, while treatment of juveniles targets the developmental plasticity during ontogeny. Since ontogenetic plasticity is often larger than behavioral flexibility in adulthood [37] we expect this treatment to be especially powerful. However, we note that this treatment during adolescence in mixed-sex groups cannot distinguish between (1) female preferences driving the differences in courtship rate via feedback on courtship and (2) male-male competition leading to differences in condition and thereby courtship rate. In contrast, the treatment with color bands during uni-sex housing (as implemented by Pariser et al. [25]) focuses on the effect of male-male interactions only and is thereby more informative about the underlying mechanism. The power of this treatment (during adulthood in uni-sex groups) could be increased by measuring courtship rates both before and after the treatment, and by looking at the within-individual change in courtship rate (longitudinal analysis). Hence, we tested both of these more powerful treatments on independent sets of birds: (1) treatment during adolescence in mixed-sex groups followed by cross-sectional analysis and (2) treatment during adulthood in uni-sex groups accompanied by longitudinal analysis. Furthermore, since research findings are often difficult to replicate across different study populations, we carried out both experiments on each of two zebra-finch populations: a domesticated European population (presumably going back to imports from Australia from the end of the 19^th^ century) and a much less domesticated population which goes back to the offspring of wild-caught birds that were imported from Australia in 1992. The latter population is genetically and morphologically indistinguishable from wild birds [38].

In the present paper we attempt to replicate the putative color band effects on male courtship rate and body mass in four independent experiments. Furthermore, we summarize all the published evidence for color band effects on courtship rate and body mass using a meta-analytic approach.

## Methods

### Ethical Note

The procedures of housing, banding, weighing and observing our study objects do not qualify as animal experimentation according to the relevant national and regional laws and are fully covered by our housing and breeding permit (# 311.4-si, by Landratsamt Starnberg, Germany). Mixed-sex housing during adolescence is a standard procedure that does not lead to high levels of aggression even when housing densities are high, apparently because birds do not yet start to form pair bonds. Hence, when birds are routinely examined after that housing period, they are typically in very good plumage condition.

### Subjects

Subjects came from two different populations of zebra finches maintained at the Max Planck Institute of Ornithology in Seewiesen, Germany (originating from populations #4 and #18 in [38]). Zebra finches have been domesticated in Europe for more than a century, and one of our populations (#18) very likely goes back to this. Hence, we refer to it as “domesticated” (D). This population last came from the University of Sheffield, UK, where it had been bred from 1985 to 2004, and has afterwards been bred for four generations (referred to as F1–F4) in Seewiesen. Subjects used for the study consist of individuals descending from F3–F3 pairings, and we refer to these as the “D_ad_” group (64 males and 61 females), because they experienced the experimental treatment as adults. Furthermore, we used offspring from a mixture of pairings across the first three generations (F1 to F3), which we refer to as the “D_juv_” group (36 males and 46 females), because they experienced the experimental treatment as juveniles.

The second population goes back to wild-caught birds whose captive-bred offspring were imported from Australia to the University of Bielefeld in 1992. This population is still closely genetically related to the wild Australian population [38], so we refer to it as “wild-type” (W). Individuals from the wild-type population are smaller (average body weight: 11.6 g) than the domesticated population (average: 17.3 g). Moreover, they are noticeably more stressed in the presence of an observer. Individuals included in the present study consist of a parental generation (“W_ad_”, 31 males and 27 females) and their offspring (“W_juv_”, 22 males and 23 females) which both arrived in Seewiesen in August 2009.

Individuals of the domesticated population were maintained under conditions as previously described [33]. They were fed *ad libitum* with a mixture of millet seeds. Water and food were changed daily, and once a week they received supplemental lettuce and vitamins. Rooms were kept at a constant temperature of 24±1°C (mean ± SD), where birds were kept under full-spectrum artificial fluorescent light at a 14∶10 h light-dark cycle. The wild-type population obtained the same food, but was housed in a glasshouse where they experienced natural light (local day lengths; experiments conducted during January – May) in addition to the above described artificial light, and a much more variable temperature (15–35°C). The more natural housing of the wild-type population was chosen because these birds had been reared in outdoor aviaries and hence are likely less well adapted to the artificial lab environment.

Individuals of the domesticated population were identified with a numbered orange aluminum band on the right leg (put on at 10 days of age). Birds of the wild-type population had been fitted with a numbered plastic band on the right leg when they were reared at the University of Bielefeld. This band was either dark green (W_ad_) or yellow (W_juv_). The dark-green color differs markedly from the light green that is normally (and here) used for manipulations of male attractiveness, but the dark green bands might nevertheless have had unintended effects on the birds. The dark green bands were therefore replaced with numbered orange plastic bands at the start of the experiment, but we cannot exclude possible long-term effects of being banded dark-green during juvenile development (W_ad_) or being reared by dark-green-banded parents (W_juv_). The yellow bands were not replaced, since we never found yellow bands to affect male attractiveness in extensive aviary experiments [34].

### Band Color Treatments

An overview of experimental treatments in comparison to the earlier study by Pariser et al. [25] is presented in Table 1. The great variety in experimental and housing conditions serves to explore the generality and robustness of color-band effects across the various conditions.

**Table 1:** Design of the four experiments carried out on males from two populations of zebra finches in comparison to the study by Pariser et al. [25].

	Domesticated population		Wild-type population		Pariser et al. [25]
	D_ad_	D_juv_	W_ad_	W_juv_	
Stage of the development	adult	juvenile	adult	juvenile	adult
Housing during color band treatment (width×depth m)(a)	cage 0.6×0.4and 2.4×0.4	cage 1.2×0.4	aviary 2×2	cage 1.2×0.4	aviary 2×3
Density (males/m^2^)	8.3 and 11.1	8.3	7.8	7.6	5.6
Duration of treatment (days)	14 and 28	74	82	89 and 101	150
Total number of males	64	36	31	22	67
- red-banded	32	18	10	11	22
- green-banded	32	18	10	11	23
- un-banded	0	0	11	1	22
Number of groups	32 and 6	9	1	6	2
Social environment	uni-sex	mixed-sex	uni-sex	mixed-sexanduni-sex	uni-sex
Baseline measurement of courtship rate before treatment	yes	no	yes	no	no

(a) Cages were 45 cm high, aviaries were 2 m high.

#### Experiment W_juv_

At 57±7 days of age (mean ± SD; i.e. on the day of arrival of these birds at Seewiesen), males from the W_juv_ group (N = 23) were randomly assigned a band color and put into groups of four males housed together with four females. Males received either a red or a green band on each leg, with the numbered yellow band remaining on the right leg, placed above the red or green band. Each group contained two green-banded and two red-banded males (five groups in total). Out of the individuals remaining from the W_juv_ pool, we constituted a sixth group of three females and three males (one green-banded, one red-banded, and one un-banded male). In total these were six groups containing 11 green and 11 red-banded males. When all the birds had reached adulthood (here at 146±6 days of age) we separated the sexes by splitting the groups of eight into uni-sex groups of four, with males still wearing their color bands up until directly before they were tested for their courtship rate and body mass at the age of 247±7 days.

#### Experiment D_juv_

When individuals from the D_juv_ group were nutritionally independent from their parents (35 days of age), we randomly assigned the males (N = 36) a band color and put them into groups of four color-banded males together with four un-banded females. The four males in a group were either all red-banded (N = 3 groups), all green-banded (N = 3 groups), or mixed with two being red-banded and two green-banded (N = 3 groups, called “mixed groups”). Hence, in total the nine groups contained 18 green and 18 red-banded males. When all the birds had reached adulthood (here at 109±5 days of age; mean ± SD) we removed the color bands and randomly allocated males into groups of two until they were tested for courtship rate and body mass four days later (at 113±5 days of age).

#### Experiment W_ad_

When the adult birds of the wild-type population arrived at Seewiesen, we housed them in two mixed-sex aviaries (with former breeding pairs staying together). About six month later, we acclimated the males (N = 31) to cages for behavioral testing (in groups of three, and separated from their females) for one week, and then tested them for their base-line courtship rate and measured their mass (at 438±19 days of age; mean ± SD). After that, males were randomly assigned to one of three treatments (10 red-banded, 10 green-banded, and 11 un-banded). These were then housed without females in one aviary for a period of 82 days. Finally, birds were acclimated again for one week to the test cages (housed in groups of three, with one male from each treatment still wearing the color bands) before taking the second courtship rate and body mass measurement.

#### Experiment D_ad_

Males from the D_ad_ group (N = 64) had been reared in mixed-sex peer groups until reaching adulthood at 115±11 days of age (mean ± SD). Then they were randomly assigned into one of 32 groups (each consisting of only two males housed in a cage) before testing them for their base-line courtship rate and body mass (at 123±11 days of age). Then, we randomly banded always one of the two males with a red and the other with a green band (32 green-banded, 32 red-banded males). We kept them in the same housing conditions for a treatment period of 14 days, after which we took a second courtship rate and body mass measurement (at 137±11 days of age). To explore effects of group size on band color effects, we next randomly assigned them into larger groups of 10–11 males (two groups of 10, four groups of 11) while switching band color for half of the males. These groups consisted of an about equal mix of red and green-banded males, but due to an assignment error there were 33 green-banded and 31 red-banded males in total. We kept them under this condition for a period of 28 days, after which we took the third courtship rate and body mass measurement (at 165±11 days of age).

### Courtship Rate and Body Mass Measurements

Color bands were always removed before behavioral testing. We measured male courtship rate during pair-wise 5-minute encounters with an unfamiliar female (from the same population and of about same age) in a standard housing cage (40 cm×60 cm and 45 cm high) as previously described [33]. Courtship rate was measured from video recordings as the number of seconds a male sings towards the female (directed song). These measurements were square-root transformed to approach normality [33]. Measurements of courtship rate were taken by two different persons, one for each population. Each male was tested with two different females (e.g. female A on day 1 and female B on day 2) on two consecutive days (between 09∶00 and 17∶30 h) and the two measurements were averaged (after transformation). The number of stimulus females used was about the same as the number of males tested in each experimental group (see numbers above), so females were re-used about as often as males were tested. In the largest of our data sets (D_ad_), female identity did not influence male courtship rate (variance component estimated to be zero), and female responsiveness (scored as previously described [33]) did not appear to influence it either (*p* = 0.83), both of which is line with earlier findings from such experimental setups [33], [34].

Measurements of body mass (to the nearest 0.1 g) were always made together with the courtship rate measurements, and always on the second test day right after the behavioral test. Inadvertently, in experiment D_ad_, body mass measurements of 20 out of 64 males were taken only with a delay of one week. Exclusion of this data did not affect the results (see below).

### Statistics

In the experimental groups W_ad_ and D_ad_, males were fully mature before the treatment began, and so it was possible to measure adult body mass and courtship rate before and after the treatment (longitudinal analysis). We compared within-individual changes in courtship rate and body mass between the treatments groups. In contrast, in the W_juv_ and the D_juv_ groups we compared the mean values after treatment between the two treatment groups (cross-sectional analysis). In the D_juv_ experiment we also included in the model the type of treatment (uni-color, i.e. red only or green only, versus mixed-color treatment). In the second part of the D_ad_ experiment, where six groups of 10–11 males each were formed, we also tested for a possible interaction term (color treatment by group identity) influencing body mass and courtship rate. This was done to test the idea that color effects might be arbitrary with respect to the color *per se*, but might arise from group dynamics leading to different effects in each group.

We analyzed the data with mixed models using the lme function from the nlme package in R 2.7.0 (R Development Core Team). These models allow us to reduce problems with pseudoreplication (non-independence of data points due to several confounding random effects). The number of potentially important random effects on male body mass and courtship rate is large (e.g. family identity, peer group identity, cage identity during measurement, identity of stimulus female in courtship tests). However, quantitative genetic analyses of a much larger data set on the domesticated population show that family identity (comprising genetic and maternal effects) is the only noteworthy factor that causes non-independence of data points [34]. Hence, all models were fitted while controlling for the males’ family identity as a random effect irrespective of significance. Residuals from the models were explored graphically to check for normality. In the Figures we always show parameters estimates and their standard errors from the mixed models, while in the text we report raw means and standard deviations. All statistical tests are two-tailed; and α was set to 0.05. Power analyses were carried out using G*Power 3.1.2 (F. Faul, Kiel, Germany).

### Meta-analysis

The total number of relevant published studies (N  = 7, see below) was too small to require a formal review protocol. They were identified by a Web of Knowledge (covering 1864–2011) search for “zebra finch* and (color* or colour*) and (band* or ring*)” which yielded 77 hits (on 29^th^ Nov 2011). Twenty seven of those actually represented studies reporting effects of red and green bands in the zebra finch, and an additional four studies were identified via forward and backward search through citations, yielding a total of 31 relevant studies (see Table S1). Among those 31 studies we found four studies presenting data on male courtship rate. These are the previously mentioned experiment by Pariser et al. [25] (data extracted from their Fig. 2 using the software Engauge Digitizer V. 4.1, http://digitizer.sourceforge.net), two experiments (on separate sets of birds housed in different group sizes) reported by Gleeson [39], and two experiments (on the same set of birds but with colors swapped among males) reported by Ratcliffe and Boag [24] (data taken from their Table 1B). We further included the study by Burley et al. [14] who reported rates of extra-pair courtship behavior by red- and green-banded males. While Pariser et al. [25], Gleeson [39], and the present study focus on courtship rate of unpaired males in standardized tests, the study by Ratcliffe and Boag [24] reports courtship of a mix of paired and unpaired males in socially complex aviary conditions, and Burley et al. [14] report only extra-pair courtships in such aviary conditions. Although the latter two studies may not be fully comparable to the others, we include them for the sake of statistical conservatism (since they report higher courtship rates by red-banded males, and we prefer to have all positive evidence included). We did not include data about color band effects on the rate of undirected singing [24], [40] since courtship and undirected singing are two very distinct behaviors with different functions and different proximate control mechanisms [41].

**Figure 1:**
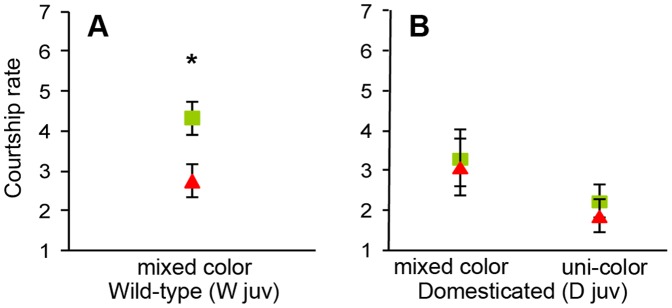
Mean ± SE of courtship rate (square-root transformed seconds of song) recorded after manipulating males with color bands during the juvenile period. All males were housed in groups of four males with four females during this period, and within each experimental group the four males were either all wearing the same color bands (uni-color; i.e. all green or all red) or there were two males of each color (mixed color). Symbol colors represent the color assigned during the experiment. (A) shows the data for the wild-type and (B) for the domesticated population. The asterisk denotes a significant treatment effect.

We also found four studies reporting color band effects on male body mass. Zann [16] presents body mass of red- and green-banded males in the wild at the start and at the end of the breeding season, and we entered these as two estimates despite their non-independence. Cuthill et al. [23] report different effects of color bands on body mass depending on the time of day (dusk versus dawn), so we also include these as two estimates. From the latter study we extracted the data from Figure 1a (using Engauge Digitizer), and averaged the values (means and SEs) for red and green-banded males across the 20 days of the study period. For both these studies our analyses are cross-sectional (like for all the above studies on courtship rate). For two studies on body mass we were able to use longitudinal data, namely Pariser et al. [25] and Schuett and Dall [7]; in the latter case we obtained the raw data from the Authors.

In the case of cross-sectional analyses we took the mean trait value (courtship rate or body mass) for both, red- and green-banded males, together with the respective standard errors (SE) and sample sizes (N) for those means, from which we calculated the within-group between-individual standard deviations (SD) in trait values. In one case [16] we estimated SDs roughly from interquartile ranges by dividing the latter with 0.67, since 50% of the data points are expected to fall within ±0.67 SD. Effect size (Cohen’s *d*, [42]) was calculated as the mean trait value (Y) of the red-banded males minus that of the green-banded males, divided by the mean of the SDs of the two groups (*d* = (Y_red_-Y_green_)/((SD_red_+SD_green_)/2)). The SE of *d* was calculated as SE_d_ = (SE_red_^2^+SE_green_^2^)^0^.^5^/((SD_red_+SD_green_)/2).

In the case of longitudinal analyses, the trait value was the within-individual change in courtship rate or mass (i.e. value after treatment minus before treatment), rather than courtship rate or mass *per se*. Accordingly, SEs and SDs refer to the between-individual variation in these changes. We combine both cross-sectional and longitudinal effect size estimates in one meta-analysis because of the overall sparseness of data, and because of no apparent difference in effect sizes between the two approaches. We use the statistics package ‘rmeta’ implemented in R 2.10.1 (R Development Core Team) to obtain an average effect size estimate with its 95% confidence interval, as well as an estimate of heterogeneity variance.

For every experiment we also recorded the housing density (in males per square meter; except for singly housed males [39] or males in the wild [16]) and correlated it with the estimated effect size on courtship rate and body mass (Spearman rank correlation). This was done to examine whether increased male-male competition due to increased housing densities would lead to greater effects of color bands on male traits (i.e. positive effects of red bands).

For a meta-analysis it is important to get from each study the parameter estimates (i.e. trait values of the red and the green-banded group) together with their correct standard errors. None of the studies controlled for any potentially confounding random effect (like genetic relatedness of individuals), so the standard errors might be slightly anticonservative, which might inflate the heterogeneity variance (between-study variance).

**Figure 2:**
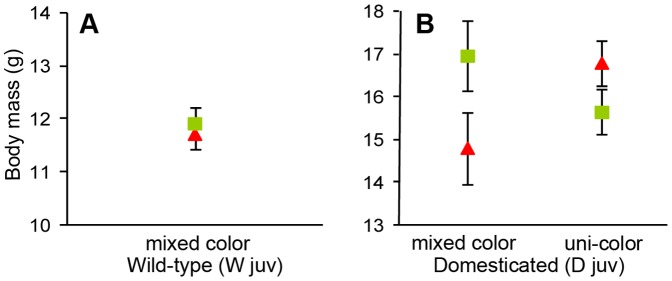
Mean ± SE of body mass measured after manipulating males during the juvenile period. All males were housed in groups of four males with four females during this period, and within each experimental group the four males were either all wearing the same color bands (uni-color; i.e. all green or all red) or there were two males of each color (mixed color). Symbol colors represent the color assigned during the experiment. (A) shows the data for the wild-type and (B) for the domesticated population.

A potential criticism of our simplified approach of comparing group means with their SEs (like in a two-sample t-test) might be that we lose power compared to more sophisticated mixed-effect models fitted in some of the original publications. The maybe most striking discrepancy in p-values obtained by different methods concerns the color band effect on courtship rate in the Pariser et al. study [25]. A two-sample t-test between the mean courtship rates of red-banded and green-banded males, with the respective SEs indicated in Figure 2 of that study yields p = 0.057 (t_36_ = 1.97; calculated using R 2.10.1). In contrast, Pariser et al. [25] report p = 0.001 from their mixed-effect model. However, since treatments were not swapped among males, the two approaches should actually yield very similar p-values. Judging from the degrees of freedom reported in the paper (df_error_  = 132), it seems that the repeatedly (4 times) measured males (n  = 47) were not nested within their color band treatments, leading to inflated significance due to pseudoreplication. Moreover, in the original analysis, the dependent variable (courtship rate) was used as a criterion to exclude 11 out of 58 males from the data set. These were the males with the lowest courtship rate, i.e. they did not display to any of the four test females. Problematically, more red-banded males (n  = 4) than green-banded males (n  = 2) were among the excluded birds; this means that the courtship rate of red-banded males was biased upwards to a greater extent than that of green-banded males. For our analysis, we conservatively assume that the males with courtship rate of zero were included in the mean values shown in Figure 2 of their paper, from which we took the estimates. The opposite assumption would further reduce the effect size obtained from that study.

## Results

### Experiment W_juv_

Contrary to our prediction, males from the wild-type population (W_juv_) that had worn red bands during juvenile development and early adulthood sang significantly less than males who had worn green bands (red: mean±SD  = 2.76±1.74 square-root transformed seconds of song, n = 11; green: mean±SD  = 4.35±0.76, n = 11; effect size d = −1.27, LMM: F_1,8_ = 7.65, *p* = 0.024, Fig. 1a). Red-banded and green-banded males did not differ in their body mass (red: mean±SD  = 11.75±0.87 grams, n = 11; green: mean±SD  = 11.95±1.07, n = 11; effect size d = −0.21, LMM: F_1,8_ = 0.26, *p* = 0.62, Fig. 2a).

### Experiment D_juv_

In the uni-color housing treatment, where all males in the cage wore the same band color, red-banded males did not differ in their courtship rate from the green-banded males at the end of the experiment (red: mean±SD  = 1.87±1.43, n = 12; green: mean±SD  = 2.22±1.38, n = 12; effect size d = −0.25, LMM: F_1,8_ = 0.41, *p* = 0.54, Fig. 1b). Red-banded males were only non-significantly heavier than green-banded males (red: mean±SD  = 16.8±2.12, n = 12; green: mean±SD  = 15.65±1.48, n = 12; effect size d = +0.64, LMM: F_1,8_ = 2.37, *p* = 0.16, Fig. 2b).

In the mixed-color housing treatment, where each cage held two males of each color, red-banded males did not differ in their courtship rate from the green-banded males (red: mean±SD  = 3.10±2.14, n = 6; green: mean±SD  = 3.32±1.26, n = 6; effect size d = −0.13, LMM: F_1,3_ = 0.05, *p* = 0.84, Fig. 1b). There was a weak tendency for red-banded males to be lighter than green-banded males (red: mean±SD  = 14.87±2.01, n = 6; green: mean±SD  = 17.00±2.00, n = 6; effect size d = −1.06, LMM: F_1,8_ = 2.37, *p* = 0.16, Fig. 2b).

Individuals from the mixed-color groups had a tendency to sing more than individuals from uni-color groups (mixed: mean±SD  = 3.20±1.71, n = 12; uni-color: mean±SD  = 2.04±1.39, n = 24; LMM: F_1,15_ = 4.18, *p* = 0.059, Fig. 1b). Overall, there was no main effect of the color manipulation (*n* = 36, F_1,14_ = 0.01, *p* = 0.93) or the group composition (uni-color vs. mixed color; *n* = 36, F_1,14_ = 0.191, *p* = 0.67) on body mass. However, there was a significant but unforeseen interaction effect between these two factors (color treatment × group-composition interaction, *n* = 36, F_1,14_ = 6.06, *p* = 0.027, Fig. 2b).

### Experiment W_ad_

**Figure 3:**
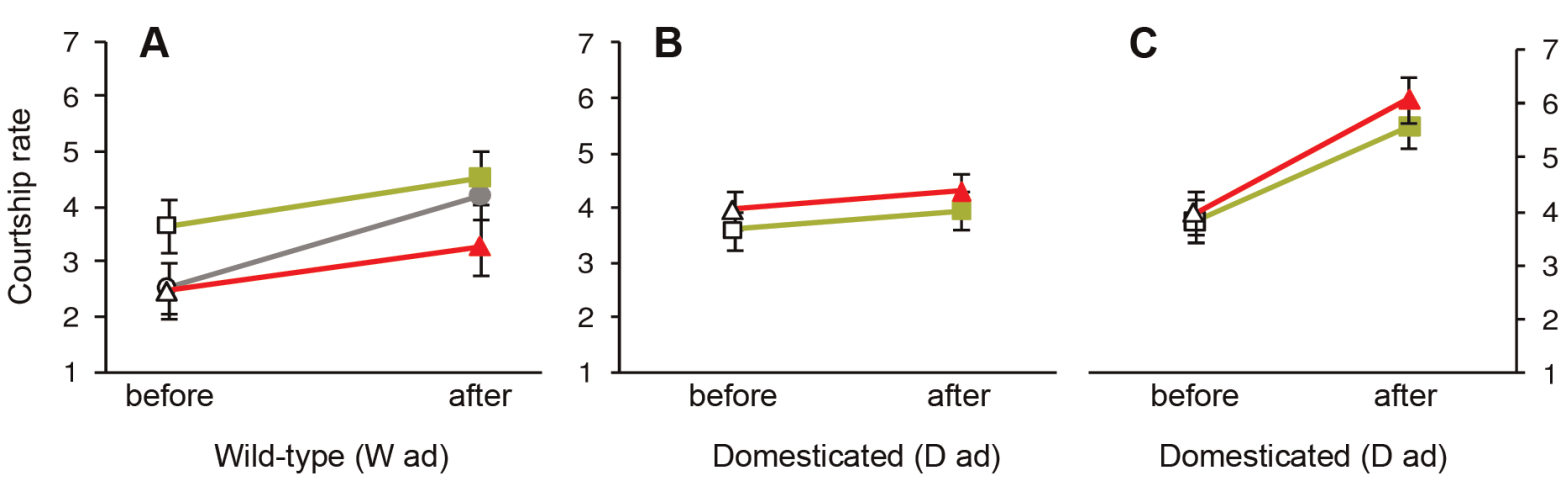
Mean ± SE of male courtship rate (transformed) before and after manipulating males by adding color bands during adulthood. Filled symbols indicate the color of the band that males were allocated and empty symbols refer to the measurement before color bands were added. (A) Males from the wild-type population (W ad), maintained in one large group containing 10 red-banded males (red symbols), 10 green-banded males (green) and 11 un-banded control males (grey); (B) males from the domesticated population (D ad), maintained in groups of two (one red-banded, one green-banded); (C) males from the domesticated population (D ad), changing from the group size of two (“before” shows the same data as the “after” in (B) but with different grouping of individuals) to groups of 10 or 11 males (with about equal numbers of red-banded and green-banded males).

**Figure 4:**
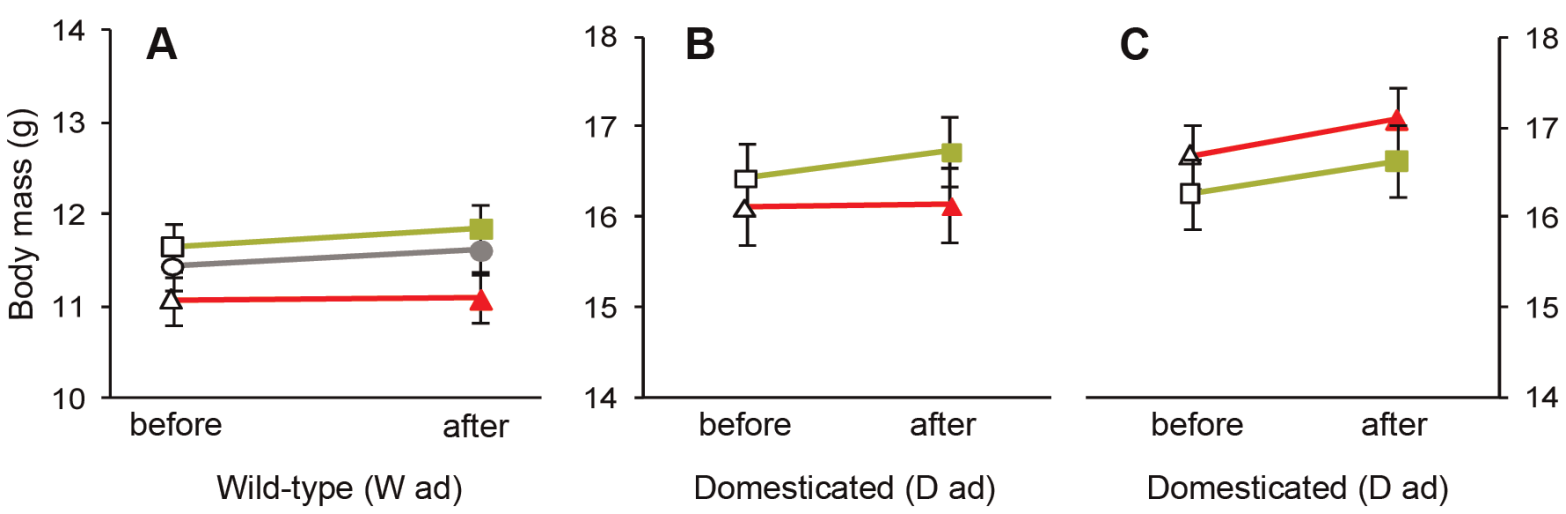
Mean ± SE of male body mass before and after manipulating males by adding color bands during adulthood. Filled symbols indicate the color of the band that males were allocated and empty symbols refer to the measurement before color bands were added. (A) Males from the wild-type population (W ad), maintained in one large group containing 10 red-banded males (red symbols), 10 green-banded males (green) and 11 un-banded control males (grey); (B) males from the domesticated population (D ad), maintained in groups of two (one red-banded, one green-banded); (C) males from the domesticated population (D ad), changing from the group size of two (“before” shows the same data as the “after” in (B) but with different grouping of individuals) to groups of 10 or 11 males (with about equal numbers of red-banded and green-banded males).

Red-banded males of the W_ad_ group did not differ significantly in their change of courtship rate (after minus before treatment) from green-banded males or from un-banded males (red: mean±SD  =  +0.82±0.81, n = 10; green: mean±SD  =  +0.88±1.70, n = 10; un-banded: mean±SD  =  +1.68±1.54, n = 11; LMM: F_2,11_ = 1.22, *p* = 0.33, Fig. 3a; effect size red vs. green d = −0.05). However, it may be noteworthy that 25 out of 31 males increased their courtship rate (paired t-test: t_30_ = 4.48, *p* = 0.0001). Treatment groups did also not differ in their body mass changes during this experiment (red: mean±SD  =  +0.01±0.60, n = 10; green: mean±SD  =  +0.21±0.37, n = 10; un-banded: mean±SD  =  +0.18±0.57, n = 11; LMM: F_2,11_ = 0.35, *p* = 0.71, Fig. 4a; effect size red vs. green d = −0.41).

### Experiment D_ad_

In the D_ad_ group of birds, red-banded males did not change their courtship rate differently from green-banded males, neither when they were housed in groups of two males (red: mean±SD  =  +0.33±1.32, n = 32; green: mean±SD  =  +0.37±1.44, n = 32; effect size d = −0.03, LMM: F_1,30_ = 0.01, *p* = 0.94, Fig. 3b), nor when housed in groups of 10 or 11 males (red: mean±SD  =  +1.85±1.51, n = 31; green: mean±SD  =  +1.64±1.54, n = 33; effect size d = +0.14, LMM: F_1,30_ = 0.36, *p* = 0.55, Fig. 3c). We note that 58 out of 64 males increased their courtship rate when moved from groups of two to groups of 10 or 11 (paired t-test: t_63_ = 9.19, *p*<0.0001). In contrast, during the first part of the experiment when males remained in groups of two, courtship rate did not change (paired t-test: t_63_ = 1.54, *p* = 0.13). Treatment groups did also not differ in their body mass changes during this experiment, neither when they were housed in groups of two males (red: mean±SD  =  +0.03±0.93, n = 32; green: mean±SD  =  +0.31±0.82, n = 32; effect size d = −0.31, LMM: F_1,30_ = 1.57, *p* = 0.22, Fig. 4b), nor when housed in groups of 10 or 11 males (red: mean±SD  =  +0.36±1.29, n = 31; green: mean±SD  =  +0.28±1.11, n = 33; effect size d = +0.07, LMM: F_1,30_ = 0.06, *p* = 0.80, Fig. 4c). This last result did not change if we excluded body mass data that were taken only a few days after the courtship rate measurements (groups of two: F_1,19_ = 2.71, *p* = 0.12; groups of 10 or 11: F_1,19_ = 0.24, *p* = 0.63). In the second part of the experiment, when six groups of 10–11 males were formed, there was also no indication of a group-identity by color-treatment interaction affecting changes in either courtship rate (F_5,52_ = 0.64, *p* = 0.67) or body mass (F_5,52_ = 0.43, *p* = 0.82).

### Meta-analysis

**Figure 5:**
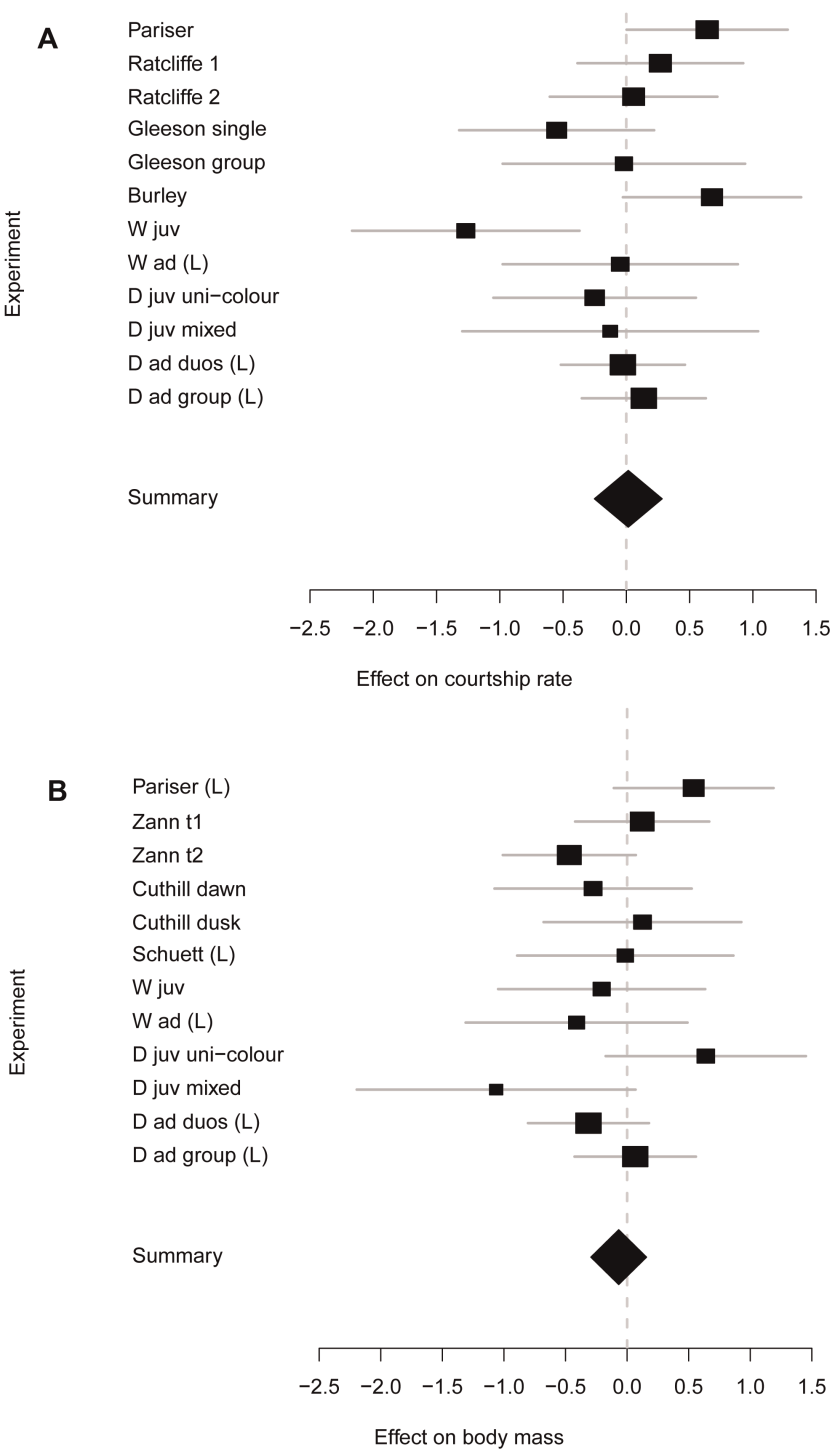
Effect sizes (d ±95% CI) of the color band treatments on (A) male courtship rate and (B) male body mass. A positive effect size refers to red-banded males obtaining higher courtship rate or mass compared to green-banded males. For individual experiments, the size of the square reflects sample size, and for the overall estimate the diamond marks the mean and the 95% CI. Longitudinal analyses as marked with (L); the remainder are cross-sectional analyses. The experiments in (a) refer to Pariser et al. [25], Ratcliffe and Boag [24] inexperienced birds (1) and experienced birds (2), Gleeson [39] individual housing and group housing, Burley et al. [14], wild-type population (W) of the present study, domesticated population (D) of the present study, juvenile (juv) and adult (ad) birds, with housing in uni-color and mixed color groups, and housing in duos or larger groups; (b) Zann [16] at the start (t1) and end (t2) of the breeding season, Cuthill et al. [23] with mass measured at either dawn or dusk, Schuett and Dall [7], and others as above.

The combined effect size of color bands on male courtship rate was estimated at d = 0.016 (95% CI -0.252–0.283). Twelve effect size estimates from six different populations contributed to this overall estimate (Fig. 5a). Effect size was unrelated to male housing density (r_s_ = −0.51, n = 11, p = 0.11, trend against the prediction). Heterogeneity variance among estimates was 0.085, which was still short of significance (*p* = 0.075).

The overall effect of color bands on body mass was even against the prediction of red males being heavier (d = −0.068), but the 95% CI also includes small positive values (−0.294–0.157). This was also based on 12 estimates from six populations (Fig. 5b). Effect size was unrelated to male housing density (r_s_ = −0.06, n = 10, p = 0.88). Heterogeneity variance among estimates was modest (0.035) and non-significant (*p* = 0.22).

## Discussion

The aim of this study was to assess the generality of an earlier finding, namely that red color bands have positive effects on male courtship rate and body mass compared to green bands [25]. However, none of the 12 tests (two times six tests shown in the bottom halves of Fig. 5a and 5b) yielded a significant effect or even a trend (p<0.15) in the expected direction. The only significant effect was against the expectation (higher courtship rates of green-banded males in the W_juv_ generation), and this might well be a type 1 error due to multiple testing: the probability of obtaining at least one significant effect from 12 tests is 38.7%. There also was a significant but counter-intuitive interaction term related to body mass (Fig. 2b). We anticipated that color bands would be effective in groups that contain both types of colors (mixed color), but potentially ineffective if all males wore the same color (uni-color). If the significant interaction in Fig. 2b is considered with this in mind, one might conclude that the positive trend (higher mass of red-banded males) in the uni-color treatment is due to chance (p = 0.16), and the negative trend (lower mass of red-banded males) in the mixed-color treatment is either meaningful (but against the expectation) or due to chance as well (also p = 0.16). The combination of these two trends in opposite directions leads to a formally significant interaction that does not appear biologically meaningful. The only highly significant effect was that courtship rate was strongly affected by housing conditions (Fig. 3c), but the experiment was not designed to answer whether this was due to changes in male group size, or simply an effect of time or other confounding factors.

Our failures to find band color effects on mass and courtship rate might be due to experimental shortcomings, so we first discuss a series of potential weaknesses of our study:

1. Lack of power: Only one out of four experiments was based on larger sample sizes than used in the initial Pariser et al. study [25] (Table 1). However, the joint sample size of all four experiments is quite large, and the pooled trends for both dependent variables are still against the expected direction (see Fig. 5). Also, as we argue in the Introduction, the design of our experiments seems more powerful than in the initial study.
2. Short treatment period: In three of our experiments treatment periods were shorter than in the Pariser et al. study [25] (Table 1). However, our longest treatment (W_juv_) did also not produce trends in the expected direction, but rather the opposite. Moreover, the effect of color bands on male dominance reported by Cuthill et al. [23] seemed to operate already within the timeframe of hours.
3. Pre-exposure to dark green bands in the wild-type population: The potentially greatest flaw of the present study is that the birds of the parental generation of the wild-type population (W_ad_) had been banded with numbered dark green identity bands before they and their offspring (W_juv_) arrived at Seewiesen. Having grown up with a dark-green identity band (W_ad_), or being reared by parents that wore a dark-green band (W_juv_), might potentially have long-term effects on the birds’ judgment about light green bands. To our knowledge, no such long-term effects have been reported in the literature (see also [40]), and it seems likely that imprinting effects (relevant for W_juv_) would have been detected and reported if they existed (e.g. in the extensive studies carried out by Nancy Burley). The dark green bands differ markedly from the light green bands in terms of brightness, but the overall hue is roughly similar. Hence, we cannot exclude with certainty that pre-exposure to dark green bands might have altered the birds’ judgment of light green bands. In contrast, there are no good reasons why this pre-exposure should have affected the judgment of red bands. Virtually all studies (including Pariser et al. [25]) that have reported positive evidence for band color effects in zebra finches have found males with red bands to be (judged) superior to males without bands or with bands of other colors. This effect was not observed in the W_ad_ generation, where we intentionally left 11 males un-banded for comparison. To the contrary, there was not even a trend supporting this idea: un-banded males tended to increase (non-significantly) rather than decrease their courtship rate relative to red and green-banded males (Fig. 3a). In summary, there are no strong reasons why this pre-exposure to dark green bands should invalidate our experiments on the wild-type population. In any case, exclusion of these experiments (W_juv_ and W_ad_) would not alter the conclusions of our meta-analysis.

The only significant main effect in our study was that green-banded males of the W_juv_ generation had higher courtship rates than their red-banded peers (Fig. 1a). One could speculate that this reversed pattern (in comparison to [25]) might have been due to the rearing by dark-green-banded parents. If such imprinting effects existed, one would also expect the females of that W_juv_ generation to show reversed mate-choice preferences (preferring light green over red). However, an extensive follow-up experiment using choice-chamber trials revealed that those females showed no band color preferences (no population-wide preference and no individual repeatability; unpublished data). Similarly, no band color preferences were found in earlier choice-chamber trials in our domesticated population (unpublished data). Hence, it might be that band color effects on male body mass and courtship rate only exist in those zebra finch populations where females also show a preference of red over green. The following review of the literature suggests that color band effects on mean body mass are generally absent or very small, while population-specific effects on courtship rate cannot be ruled out with certainty.

### Evidence from the Literature

For each of the dependent variables, courtship rate and body mass, we were able to find six effect size estimates from four different study populations. When combining these with our own data (six estimates from two populations) the overall effect size estimates are very close to zero, and significantly smaller than 0.3, which is conventionally considered a small effect size. Note that, to detect an effect size of d = 0.3 with 80% probability in a cross-sectional analysis, a sample size of 139 red-banded and 139 green-banded males would be required; more than used in any published zebra finch study on color band effects. In our meta-analysis, publication bias seems to play only a minor role, since only one study [25] claims positive evidence for what we here examine. The study by Cuthill et al. [23] focused on body-mass changes from dawn to dusk rather than on body mass measured once during the day. Our cross-sectional analysis of their longitudinal patterns lacks the statistical power of their analysis, but also illustrates how small the there described effect is when measured in units of between-individual SD in body mass (Fig. 5b).

In the case of body mass, there was little heterogeneity among the 12 effect size estimates, but in the case of courtship rate, heterogeneity variance was not far from reaching significance (*p* = 0.075). The latter was mostly due to the contrast between positive estimates (i.e. red-banded males courting more) from Burley [14] with *p* = 0.07 and Pariser et al. [25] with *p* = 0.057 and the negative estimate (i.e. green-banded males courting more) from the second generation of our wild-type population with *p* = 0.024. Often, such discrepancies in findings are suspected to be due to population-specific effects, like genetic drift between isolated domesticated populations [38]. This view does not seem to be supported here. Firstly, the parental generation of our wild-type population (W_ad_) yielded a very different effect estimate from the one obtained from their offspring (W_juv_; Fig. 5a). Secondly, Gleeson [39] conducted an experiment very similar to that of Pariser et al. [25], with the former using captive-bred offspring of wild-caught birds and the latter using wild-caught birds directly. Also here, the effect size estimates are very different (Fig. 5a), despite an expectation of genetic uniformity [38]. Hence, clearly more data would be required to understand whether the observed heterogeneity variance is meaningful (not just sampling error), and potentially what factors might contribute to it. One possibility is that the effects of color bands on courtship rate only exist in populations where females also show preferences for red bands over green bands. Such preferences are clearly present in the population studied by Burley [14], and Pariser et al. [25] report that females responded more often with positive signals in reaction to courtship by red-banded males (yet the scoring was not done blindly). As mentioned earlier, color band preferences were found to be absent in our two zebra finch populations (unpublished data), and nothing is known about band color preferences in the populations studied by Gleeson [39] and Ratcliffe and Boag [24]. So the hypothesis of a joint occurrence of female preferences for red bands and an effect of red bands on courtship rate might deserve further investigation.

The view that populations might differ with regard to color band effects might question the approach of lumping all 12 estimates (Figure 5) into one analysis, irrespective of the population from which they stem. We therefore also calculated mean effect sizes for each population separately using the ‘rmeta’ package. For courtship rate, mean effect sizes were non-significantly positive for each of three populations (Pariser, Ratcliffe, and Burley; i.e. one Australian and two North American populations) and non-significantly negative for each of the other three populations (Gleeson and this study; i.e. one Australian, one European, and one European population recently imported from Australia). For body mass, mean effect sizes were non-significantly positive for the Pariser population, and non-significantly negative for each of the other five populations.

Overall, there is no single study that has convincingly (i.e. repeatedly [3]) demonstrated a color band effect on either courtship rate or body mass. Considering the current mean effect size estimates, it may be the wisest to assume the complete absence of such effects. The findings of Pariser et al. [25] might well represent a false-positive result, when also considering that its statistical significance appears to hinge on pseudoreplication and data selection (see Methods). While positive color-band effects on courtship rate and body mass seem difficult to replicate, there is a series of studies convincingly demonstrating that, in some laboratories, females prefer red-banded over green-banded males (see Table S1). In this case, it would still seem highly rewarding to work out why these effects have not been found in all or most populations. A first step towards this goal would be the publication of more experiments (with positive or negative outcomes) that, apparently, have been considered not worth reporting. The second step could be the exchange of birds among laboratories with contrasting findings, to work out the extent to which differences in results are due to variation in experimental setups, rearing conditions or genetics.

## Supporting Information

Table S1 **Studies reporting color-band effects.**
(DOC)
