# Supplementary material for: No Band Color Effects on Male Courtship Rate or Body Mass in the Zebra Finch: Four Experiments and a Meta-Analysis
Source: PLoS One. 2012 Jun 7;7(6):e37785. doi: 10.1371/journal.pone.0037785 (PMC3369886; doi:10.1371/journal.pone.0037785)
Supplement: Table S1 — Studies reporting color-band effects. (DOC) [file pone.0037785.s001.doc]

Table S1. Studies reporting color band effects on a range of traits, categorized as to whether the expected main effect was found or not

| **Category** | **Trait** | **Main effect** | **Interactions** | **Population** | **Reference** |
| --- | --- | --- | --- | --- | --- |
| fitness | fitness | yes |  | Illinois | Burley [1] |
| fitness | fitness | yes |  | Illinois | Burley [2] |
| fitness | fitness: within-pair success | yes |  | Illinois | Burley [3] |
| survival | survival | yes |  | Illinois | Burley [4] |
| survival | survival | no |  | Wild | Zann [5] |
| preference | choice chamber preference | yes |  | Irvine | Burley [6] |
| preference | choice chamber preference | yes |  | Illinois | Burley [7] |
| preference | choice chamber preference | yes |  | Wild | Burley [8] |
| preference | choice chamber preference | yes |  | Irvine | Burley [9] |
| preference | choice chamber preference | yes |  | Illinois | Burley [10] |
| preference | EPCs | yes |  | Illinois | Burley [11] |
| preference | choice chamber preference | yes |  | Irvine | Burley [12] |
| preference | choice chamber preference | yes |  | Bristol | Hunt [13] |
| preference | choice chamber preference | no |  | Oxford | Jennions [14] |
| maternal | sex ratio | yes |  | Illinois | Burley [1] |
| maternal | sex ratio | yes |  | Illinois | Burley [15] |
| maternal | parental care | yes |  | Illinois | Burley [16] |
| maternal | yolk testosterone | yes |  | StAndrews | Gil [17] |
| maternal | offspring traits | no ^(a)^ | yes | StAndrews | Gilbert [18] |
| maternal | parental care | no | yes | Glasgow | Gorman [19] |
| maternal | female egg production | no | yes | StAndrews | Rutstein [20] |
| maternal | sex ratio | no |  | StAndrews | Rutstein [20] |
| maternal | yolk testosterone | no |  | StAndrews | Rutstein [20] |
| maternal | sex ratio | no ^(b)^ |  | StAndrews | Rutstein [21] |
| maternal  maternal  maternal | yolk carotenoids  daughter fecundity  son attractiveness | no  yes  no | yes | StAndrews  StAndrews  StAndrews | Williamson [22]  Gilbert [23]  Gilbert [23] |
|  |  |  |  |  |  |
|  |  |  |  |  |  |
|  |  |  |  |  |  |
| maternal | female egg production | yes |  | Wild | Zann [5] |
| maternal | sex ratio | no |  | LaTrobe | Zann [24] |
| male trait | dominance | yes |  | Bristol | Cuthill [25] |
| male trait | fat gain | yes |  | Bristol | Cuthill [25] |
| male trait | mass changes | yes |  | Bristol | Cuthill [25] |
| male trait | song rate | no | yes | Wild | Gleeson [26] |
| male trait | beak color | no | yes | Wild | Gleeson [26] |
| male trait | immunity | no | yes | Wild | Gleeson [26] |
| male trait | courtship rate | yes |  | Wild | Pariser [27] |
| male trait | mass | no ^(c)^ |  | Wild | Pariser [27] |
| male trait | dominance | no |  | Queen's | Ratcliffe [28] |
| male trait | dominance | no |  | Exeter | Schuett [29] |
| male trait | fat | no |  | Exeter | Schuett [29] |
| male trait | mass | no |  | Exeter | Schuett [29] |
| male trait | mass | no |  | Wild | Zann [5] |
| other | social learning | yes |  | StAndrews | Benskin [30] |
| other | tutor choice | no |  | StAndrews | Pearson [31] |
| other | male attention to female | yes |  | Glasgow | Royle [32] |

1. Except offspring begging behavior
2. Effect against the predicted direction
3. Effect non-significant if tested with ANOVA

**REFERENCES**

1. Burley N (1981) Sex-ratio manipulation and selection for attractiveness. Science 211: 721-722.

2. Burley N (1986) Sexual selection for aesthetic traits in species with biparental care. American Naturalist 127: 415-445.

3. Burley NT, Parker PG, Lundy K (1996) Sexual selection and extrapair fertilization in a socially monogamous passerine, the zebra finch (Taeniopygia guttata). Behavioral Ecology 7: 218-226.

4. Burley N (1985) Leg-band color and mortality patterns in captive breeding populations of zebra finches. Auk 102: 647-651.

5. Zann R (1994) Effects of band color on survivorship, body condition and reproductive effort of free-living australian zebra finches. Auk 111: 131-142.

6. Burley NT, Foster VS (2004) Digit ratio varies with sex, egg order and strength of mate preference in zebra finches. Proceedings of the Royal Society of London Series B-Biological Sciences 271: 239-244.

7. Burley N (1986) Comparison of the band-color preferences of 2 species of estrildid finches. Animal Behaviour 34: 1732-1741.

8. Burley N (1988) Wild zebra finches have band-color preferences. Animal Behaviour 36: 1235-1237.

9. Burley NT (2006) An eye for detail: Selective sexual imprinting in zebra finches. Evolution 60: 1076-1085.

10. Burley N, Krantzberg G, Radman P (1982) Influence of color-banding on the conspecific preferences of zebra finches. Animal Behaviour 30: 444-455.

11. Burley NT, Enstrom DA, Chitwood L (1994) Extra-pair relations in zebra finches - differential male success results from female tactics. Animal Behaviour 48: 1031-1041.

12. Burley NT, Foster VS (2006) Variation in female choice of mates: condition influences selectivity. Animal Behaviour 72: 713-719.

13. Hunt S, Cuthill IC, Swaddle JP, Bennett ATD (1997) Ultraviolet vision and band-colour preferences in female zebra finches, Taeniopygia guttata. Animal Behaviour 54: 1383-1392.

14. Jennions MD (1998) The effect of leg band symmetry on female-male association in zebra finches. Animal Behaviour 55: 61-67.

15. Burley N (1986) Sex-ratio manipulation in color-banded populations of zebra finches. Evolution 40: 1191-1206.

16. Burley N (1988) The differential-allocation hypothesis an experimental test. American Naturalist 132: 612-628.

17. Gil D, Graves J, Hazon N, Wells A (1999) Male attractiveness and differential testosterone investment in zebra finch eggs. Science 286: 126-128.

18. Gilbert L, Williamson KA, Hazon N, Graves JA (2006) Maternal effects due to male attractiveness affect offspring development in the zebra finch. Proceedings of the Royal Society B-Biological Sciences 273: 1765-1771.

19. Gorman HE, Arnold KE, Nager RG (2005) Incubation effort in relation to male attractiveness in zebra finches Taeniopygia guttata. Journal of Avian Biology 36: 413-420.

20. Rutstein AN, Gilbert L, Slater PJB, Graves JA (2004) Mate attractiveness and primary resource allocation in the zebra finch. Animal Behaviour 68: 1087-1094.

21. Rutstein AN, Gorman HE, Arnold KE, Gilbert L, Orr KJ, et al. (2005) Sex allocation in response to paternal attractiveness in the zebra finch. Behavioral Ecology 16: 763-769.

22. Williamson KA, Surai PF, Graves JA (2006) Yolk antioxidants and mate attractiveness in the Zebra Finch. Functional Ecology 20: 354-359.

23. Gilbert L, Williamson KA, Graves JA (2012) Male attractiveness regulates daughter fecundity non-genetically via maternal investment. Proceedings of the Royal Society of London Series B-Biological Sciences 279: 523-528.

24. Zann R, Runciman D (2003) Primary sex ratios in zebra finches: no evidence for adaptive manipulation in wild and semi-domesticated populations. Behavioral Ecology and Sociobiology 54: 294-302.

25. Cuthill IC, Hunt S, Cleary C, Clark C (1997) Colour bands, dominance, and body mass regulation in male zebra finches (Taeniopygia guttata). Proceedings of the Royal Society of London Series B-Biological Sciences 264: 1093-1099.

26. Gleeson DJ (2006) Context-dependent effect of social environment on immune response and sexual signalling in male zebra finches. Australian Journal of Zoology 54: 375-379.

27. Pariser EC, Mariette MM, Griffith SC (2010) Artificial ornaments manipulate intrinsic male quality in wild-caught zebra finches (Taeniopygia guttata). Behavioral Ecology 21: 264-269.

28. Ratcliffe LM, Boag PT (1987) Effects of color bands on male competition and sexual attractiveness in zebra finches (Poephila guttata). Canadian Journal of Zoology-Revue Canadienne De Zoologie 65: 333-338.

29. Schuett W, Dall SRX (2010) Appearance, "state," and behavior in male zebra finches, Taeniopygia guttata. Journal of Ethology 28: 273-286.

30. Benskin C, Mann NI, Lachlan RF, Slater PJB (2002) Social learning directs feeding preferences in the zebra finch, Taeniopygia guttata. Animal Behaviour 64: 823-828.

31. Pearson FD, Mann NI, Slater PJB (1999) Does leg-ring colour affect song tutor choice in zebra finches? Animal Behaviour 57: 173-180.

32. Royle NJ, Pike TW (2010) Social feedback and attractiveness in zebra finches. Behavioral Ecology and Sociobiology 64: 2015-2020.
